# First record of the genus *Wilkinsonellus* (Hymenoptera, Braconidae, Microgastrinae) from Fiji with description of three new species

**DOI:** 10.3897/zookeys.397.7195

**Published:** 2014-04-03

**Authors:** Diana Carolina Arias-Penna, Yali Zhang, James B. Whitfield

**Affiliations:** 1 Department of Entomology, 320 Morrill Hall, 505 S. Goodwin Ave., University of Illinois, Urbana, IL 61801, USA

**Keywords:** Braconidae, diversity, inventory, parasitoid wasps

## Abstract

*Wilkinsonellus* Mason is a relatively small Pantropical genus of braconid parasitoid wasps within the subfamily Microgastrinae. Most of the currently described species are from the Palaeotropics; however, previous records were absent from Fiji. Here, the first three *Wilkinsonellus* species from Fiji are described: *Wilkinsonellus corpustriacolor* **sp. n.**, *Wilkinsonellus fijienis* **sp. n.** and *Wilkinsonellus nescalpura* **sp. n.** The material was collected by Malaise traps set up in a quite variety of ecosystems (wet zone, dry zone and coastal forests) throughout the archipelago. With these records, Fiji represents the easternmost known distribution of the genus in the Indo-Pacific Region. A key to all of the currently known *Wilkinsonellus* species is included to facilitate species identification.

## Abstract (es)

*Wilkinsonellus* Mason es un pequeño género Pantropical de avispas parasitoideas en la subfamilia Microgastrinae (Hymenoptera). La mayoría de las especies descritas son Paleotropicales, aunque hasta ahora el género no había sido previamente registrado en Fiji. En esta publicación, tres nuevas especies son descritas para Fiji: *Wilkinsonellus corpustriacolor* **sp. n.**, *Wilkinsonellus fijienis* **sp. n.** y *Wilkinsonellus nescalpura* **sp. n.** Las muestras fueron colectadas mediante trampas Malaise colocadas en un gran variedad de ecosistemas (zonas húmedas, zonas secas y bosques costeros) en el archipiélago. Fiji es el registro más oriental conocido del género en la región Indo-Pacifica. Una clave que incluye todas las especies descritas de *Wilkinsonellus* es provista para facilitar la identificación de las especies.

## Introduction

*Wilkinsonellus* Mason, 1981 are parasitoid wasps within Microgastrinae (Braconidae: Hymenoptera) of Pantropical distribution. The genus currently contains 19 species (Table 1), of which three species were recently reported in the Neotropics (Arias-Penna et al. 2013). The identity of hosts parasitized by those wasps remains essentially undocumented. As all the subfamily genera, *Wilkinsonellus* possibly are koinobiont endoparasitoids of Lepidoptera that exclusively attack the larval (caterpillar) stage. The only lepidopteran host reported up to date is *Microthyris prolongalis* (Crambidae) in the Neotropical species *Wilkinsonellus alexsmithi* (Arias-Penna et al. 2013). The genus is easy to recognize from other Microgastrinae genera mainly by its characteristic propleuron (with a posterior rounded flange), the shape of the vein 1-1A on the fore wing (strongly curved, and almost touching the margin of the wing), and the shape of the petiole on tergite I (petiole 4–5 times as long as its apical width, constricted medially and deeply grooved almost to its apex) (Whitfield 1997, Long and van Achterberg 2003, Zeng et al. 2011).

**Table 1.** Checklist of the *Wilkinsonellus* species currently described

*Wilkinsonellus* species	Descriptor	Distribution	Reference
*Wilkinsonellus alexsmithi*	Arias-Penna & Whitfield, 2013	Costa Rica	Arias-Penna et al. 2013
*Wilkinsonellus amplus*	Austin & Dangerfield, 1992	Australia	Austin and Dangerfield 1992
*Wilkinsonellus daira*	(Nixon, 1965)	Papua New Guinea	Nixon 1965
*Wilkinsonellus flavicrus*	Long & van Achterberg, 2011	Taiwan	Long and van Achterberg 2011
*Wilkinsonellus granulatus*	Ahmad, Pandey, Haider & Shuja-Uddin 2005	India	Ahmad et al. 2005
*Wilkinsonellus henicopus*	(de Saeger, 1944)	Kenya and Rwanda	Nixon 1965
*Wilkinsonellus iphitus*	(Nixon, 1965)	Philippines, Taiwan	Nixon 1965, Chou 1999
*Wilkinsonellus kogui*	Arias-Penna & Whitfield, 2013	Colombia	Arias-Penna et al. 2013
*Wilkinsonellus longicentrus*	Long & van Achterberg, 2003	Vietnam	Long and van Achterberg 2003, 2011
*Wilkinsonellus masoni*	Long & van Achterberg, 2011	Vietnam	Long and van Achterberg 2011
*Wilkinsonellus narangahus*	Rousse & Gupta, 2013	Reunion Island	Rousse and Gupta 2013
*Wilkinsonellus nigratus*	Long & van Achterberg, 2011	Vietnam	Long and van Achterberg 2011
*Wilkinsonellus nigrocentrus*	Long & van Achterberg, 2011	Vietnam	Long and van Achterberg 2011
*Wilkinsonellus panamaensis*	Arias-Penna & Whitfield, 2013	Panama	Arias-Penna et al. 2013
*Wilkinsonellus paramplus*	Long & van Achterberg, 2003	Vietnam and China	Long and van Achterberg 2003, 2011; Zeng et al. 2011
*Wilkinsonellus striatus*	Austin & Dangerfield, 1992	Australia and Papua New Guinea	Austin and Dangerfield 1992
*Wilkinsonellus thyone*	(Nixon, 1965)	Philippines	Nixon 1965
*Wilkinsonellus tobiasi*	Long, 2007	Vietnam	Long 2007, Long and van Achterberg 2011
*Wilkinsonellus tomi*	Austin & Dangerfield, 1992	Australia and Papua New Guinea	Austin and Dangerfield 1992

There are no available records of *Wilkinsonellus* from Fiji, except those newly reported here. In the Palaeotropics the genus has been previously reported in Africa, South and Southeast Asia, Indonesia, Australia and Papua New Guinea (Nixon 1965, Austin and Dangerfield 1992, Long and van Achterberg 2003, 2011, Ahmad et al. 2005, Long 2007). Most, if not all, microgastrine species currently reported from Fiji (e.g. Evenhuis 2007, Prasad 2010) come from checklists published in the first two thirds of the twentieth century (Turner 1919, Fullaway 1957). As so far reported, the Fijian Microgastrinae fauna includes a total of 18 species that belong to four genera as follows: *Apanteles* [*Apanteles aganoxenae* Fullaway, 1941; *Apanteles carpatus* (Say, 1836); *Apanteles expulsus* Turner, 1919; *Apanteles heterusiae* Wilkinson, 1928; *Apanteles hyblaeae* Wilkinson, 1928; *Apanteles hymeniae* Wilkinson, 1935; *Apanteles platyedrae* Wilkinson, 1928; *Apanteles samoanus* Fullaway, 1940; *Apanteles stantoni* (Ashmead, 1904), *Apanteles tirathabae* Wilkinson, 1928]; *Cotesia* [*Cotesia glomerata* (Linnaeus, 1758); *Cotesia marginiventris* (Cresson, 1865); *Cotesia plutellae* (Kurdjumov, 1912); *Cotesia ruficrus* (Haliday, 1834)]; *Glyptapanteles* [*Glyptapanteles artonae* (Rohwer, 1926), *Glyptapanteles phytometrae* (Wilkinson, 1928), *Glyptapanteles taylori* (Wilkinson, 1928)] and *Sathon* [*Sathon belippae* (Rohwer, 1918)] (Turner 1919, Fullaway 1957, Evenhuis 2007, Prasad 2010). Inventory samples make clear that this is only a fraction of the total species found there.

This paper provides descriptions of the three new *Wilkinsonellus* species, representing the first records of this genus in Fiji and establishing this island country as the easternmost distribution of the genus in the Indo-Pacific Region so far. This revision elevates the current total of *Wilkinsonellus* species known worldwide to 22.

## Methods

The Fijian Archipelago is one of the most unique island groups within the Indo-West Pacific region, characterized by an exceptionally high species richness documented mainly in marine ecosystems (Bromfield and Pandolfi 2012). Fiji consists of over 300+ named islands of which Viti Levu and Vanua Levu are the largest islands, followed by the mid-sized Taveuni, Kadavu, Ovalau, Gau and Koro, the remainder being small islands (Evenhuis and Bickel 2005). Biogeographically the archipelago is very interesting due to the proximity to other major Pacific island groups such as the Samoan Archipelago to the northeast, Tonga to the east, Vanuatu to the west and New Caledonia to the southwest. Fiji has a warm, humid tropical maritime climate, with moisture brought by the south-east winds. The wet zones are found on the windward side of the islands, while the dry zones are on the leeward (Bickel 2008). The endemic fauna from Fiji is concentrated almost exclusively in terrestrial ecosystems; however many of the tropical forests have been cleared by loggers and converted to plantations [e.g. copra, ginger, tropical fruits, cocoa and rice] (Department of Environment 2010).

The material reviewed for this revision comprises specimens collected under the three-years project “The Fiji Terrestrial Arthropod Survey” (http://hbs.bishopmuseum.org/fiji/) funded by the National Science Foundation (DEB-0425790) and the Schlinger Foundation. The project was conducted by Dr. Neal L. Evenhuis (Bernice Pauahi Bishop Museum, Hawaii), and Dr. Daniel J. Bickel (Australian Museum, Australia). Between 2005 to 2008, the survey of terrestrial arthropods in the Fijian islands collected about 700,000 specimens covering the wet zone (lowland rain forest, montane rain forest and cloud forest), dry zone, and coastal forests (limestone forest and lowland moist forest) in twelve islands of the archipelago (i.e. Gau, Koro, Kadavu, Lakeba, Macuata, Moala, Ovalau, Taveuni, Vanua Levu, Viti Levu, Yadua Taba and Yasawa).

Two to five Malaise traps were set up at each site; all traps were monitored regularly and samples were collected each 12 days. Specimens were preserved in 95% ethanol. A team of parataxonomist sorted and processed all the material at the Ministry of Forestry laboratory facility, Colo-i-Suva, Fiji. Afterwards, all samples were first sent to Bernice Pauahi Bishop Museum, Hawaii, and after sorting were subsequently shipped to different specialists around the world. Part of the Ichneumonoidea samples were sent to the University of Illinois Urbana-Champaign, UIUC, USA (James B. Whitfield Lab).

### Morphology and taxonomic characters

At UIUC, Microgastrinae were sorted from the other Ichneumonoidea. Later on, *Wilkinsonellus* specimens were separated from the rest of Microgastrinae following a key to the genera (Whitfield 1997). A previous key to species (Long and van Achterberg 2011) and the original species descriptions (Nixon 1965, Austin and Dangerfield 1992, Long and van Achterberg 2003, Ahmad et al. 2005, Long 2007, Long and van Achterberg 2011, Arias-Penna et al. 2013) were used in order to confirm if the specimens matched with species previously described.

For easy manipulation and to avoid specimen fragmentation during handling, samples were soaked with Hexamethyldisilazane HMDS, [(CH_3_)_3_Si]_2_NH for 1 hour at room temperature and afterwards pinned (point mounted). Specimens treated with this chemical can be subsequently processed for DNA extraction and amplification (Heraty and Hawks 1998).

The cuticular sculpturing terminology follows Harris (1979), and morphological terms for body structures as well as venation follow Sharkey and Wharton (1997). Species descriptions are based on the holotype female, and infraspecific variation for the three species is reported when possible. Photos were taken with a Leica DFC425 digital microscope camera attached to a Leica M205 stereomicroscope (Wetzlar, Germany). The LAS (Leica Application Suite) multifocus module integrated within the Leica microscope was used for taking the pictures. The stack of images at different focus positions was processed with Zerene Stacker version 1.04 (http://zerenesystems.com/cms/stacker) and post-processed with Adobe Photoshop CS5.

Holotypes are deposited in the Fiji National insect Collection in Suva, Fiji and paratypes in Bernice Pauahi Bishop Museum, Honolulu, Hawaii, USA and California Academy of Sciences, San Francisco, California, USA.

The following are the abbreviations used in the text:

ATM = axillary trough of metanotum; ATS = axillary trough of scutellum; BM = medioposterior band of metanotum; BS = medioanterior band of scutellum; L = lunule of scutellum; MPM = medioposterior pit of metanotum; OOL = ocular ocellar line: the shortest distance between posterior ocellus and adjacent compound eye margin; POL = posterior ocellar line: the shortest distance between the posterior ocelli; PRM = posterior rim of metanotum.

## Results

### Wilkinsonellus

Mason, 1981

http://species-id.net/wiki/Wilkinsonellus

#### Type species.

*Apanteles iphitus*, Nixon 1965

#### Diagnosis.

**Figure 1.**
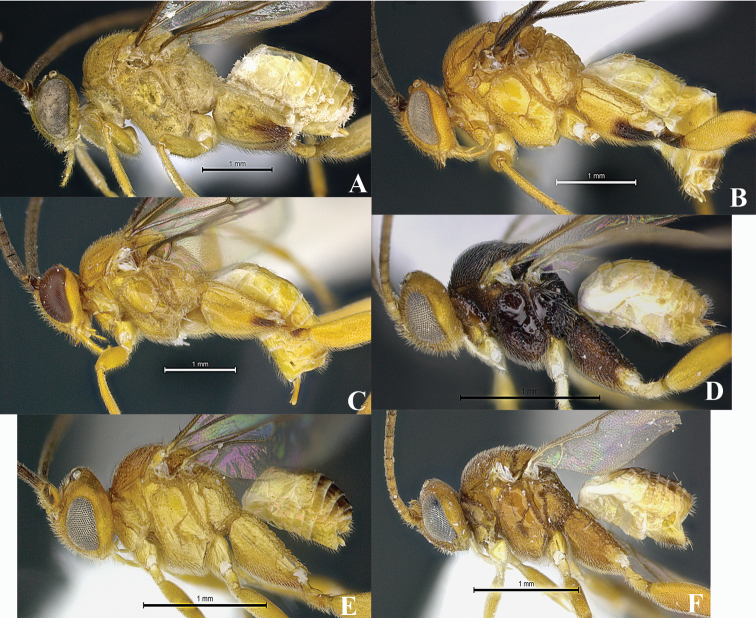
Habitus *Wilkinsonellus* species. **A** Female, *Wilkinsonellus alexsmithi* Arias-Penna & Whitfield, 2013 **B** Male, *Wilkinsonellus kogui* Arias-Penna & Whitfield, 2013 **C** Female, *Wilkinsonellus panamaensis* Arias-Penna & Whitfield, 2013 **D** Female, *Wilkinsonellus corpustriacolor* Arias-Penna, Zhang & Whitfield, sp. n. **E** Female, *Wilkinsonellus fijiensis* Arias-Penna, Zhang & Whitfield, sp. n. **F** Female, *Wilkinsonellus nescalptura* Arias-Penna, Zhang & Whitfield, sp. n.

**Figure 2.**
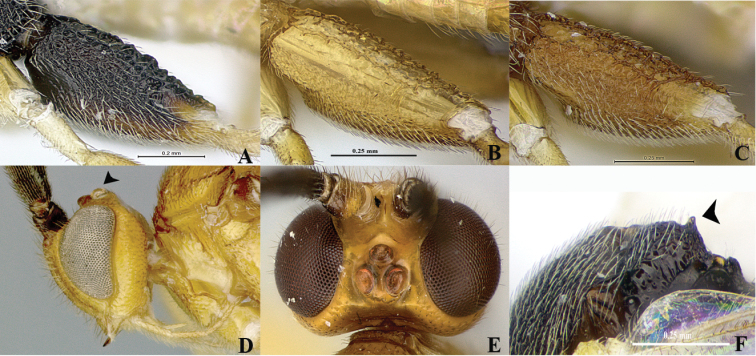
Body structures *Wilkinsonellus* species. **A–C** Female, hind coxa **A** *Wilkinsonellus corpustriacolor* Arias-Penna, Zhang & Whitfield, sp. n. **B** *Wilkinsonellus fijiensis* Arias-Penna, Zhang & Whitfield, sp. n. **C** *Wilkinsonellus nescalptura* Arias-Penna, Zhang & Whitfield, sp. n. **D–E** Head **D** *Wilkinsonellus kogui* Arias-Penna & Whitfield, 2013, lateral view. **E** *Wilkinsonellus panamaensis* Arias-Penna & Whitfield, 2013, dorsal view **F** Scutellum with apical spine, *Wilkinsonellus corpustriacolor* Arias-Penna, Zhang & Whitfield, sp. n.

**Figure 3.**
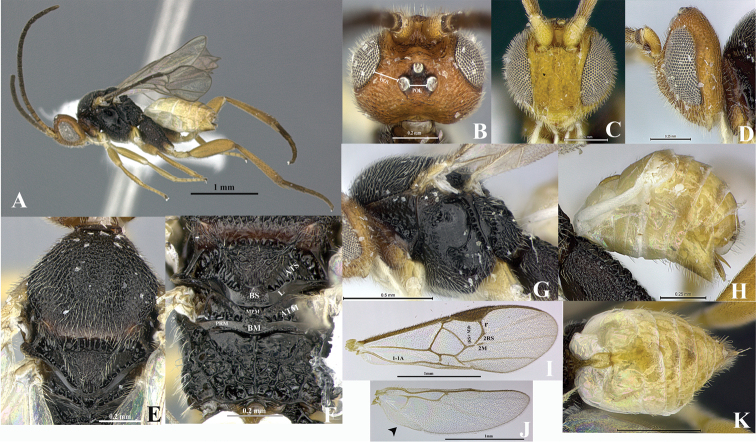
Female, *Wilkinsonellus corpustriacolor* Arias-Penna, Zhang & Whitfield, sp. n. **A** Habitus **B–D** Head **B** Dorsal view **C** Frontal view **D** Lateral view **E** Mesoscutum, dorsal view **F** Metanotum & Propodeum, dorsal view **G** Mesosoma, lateral view **H** Metasoma, lateral view **I–J** Wings **I** Fore **J** Hind **K** Metasoma, dorsal view.

**Figure 4.**
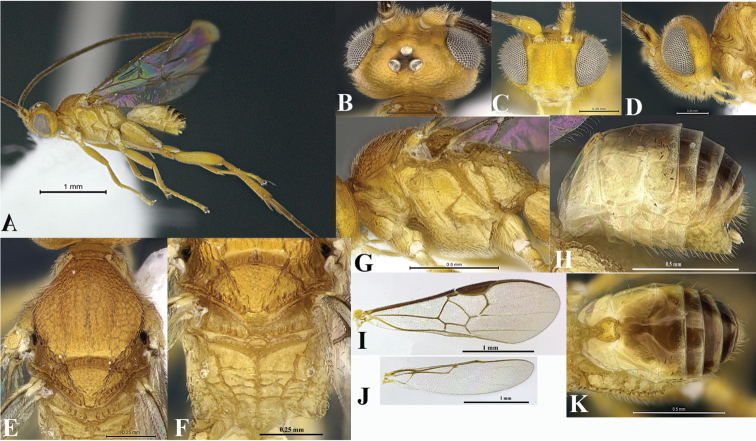
Female, *Wilkinsonellus fijiensis* Arias-Penna, Zhang & Whitfield, sp. n. **A** Habitus **B–D** Head **B** Dorsal view **C** Frontal view **D** Lateral view **E** Mesoscutum, dorsal view **F** Metanotum & Propodeum, dorsal view **G** Mesosoma, lateral view **H** Metasoma, lateral view **I–J** Wings **I** Fore **J** Hind **K** Metasoma, dorsal view.

**Figure 5.**
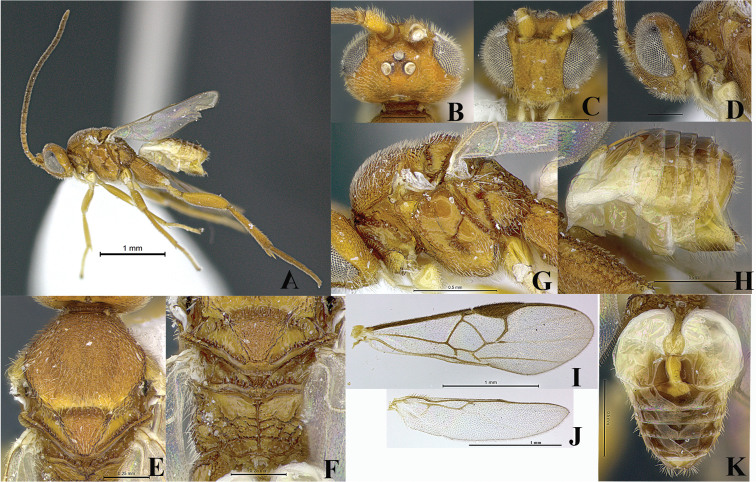
Female, *Wilkinsonellus nescalptura* Arias-Penna, Zhang & Whitfield, sp. n. **A** Habitus **B–D** Head **B** Dorsal view **C** Frontal view **D** Lateral view **E** Mesoscutum, dorsal view **F** Metanotum & Propodeum, dorsal view **G** Mesosoma, lateral view **H** Metasoma, lateral view **I–J** Wings **I** Fore **J** Hind **K** Metasoma, dorsal view.

*Wilkinsonellus* is distinguishable from other Microgastrinae genera by the following combination of traits: body coloration largely yellowish or brown-yellow (Figs 1A–F); propleuron with a posterior flange (Figs 1A–F, 2D, 3G, 4G, 5G); scutellum sculptured medio-posteriorly and often with subapical carina (Figs 3F, 4F, 5F); lunules of scutellum narrow (Figs 3F, 4F, 5F); fore wing with vein r-m absent and vein 1-1A strongly curved (Figs 3I, 4I, 5I), lying very close to posterior margin of the wing (Long and Achterberg 2003); propodeum with a median carina (Figs 3F, 4F, 5F), spiracles completely or partially surrounded by carinae; tergite I (Figs 3K, 4K, 5K) with petiole 4–5 times as long as its apical width, more or less constricted medially and deeply grooved almost to apex (Zeng et al. 2011); median longitudinal area of metasomal tergite II slightly raised, usually poorly delimited (Figs 3K, 4K, 5K), tergite II as long as tergite III, both smooth (Whitfield 1997); hind coxa enlarged (Figs 1A–F, 3A, 4A, 5A), rarely short except in *Wilkinsonellus flavicrus* (Long and Achterberg 2011); ovipositor sheaths short (Figs 3H, 4H, 5H) (Whitfield 1997).

#### Key to species of the genus *Wilkinsonellus* Mason, 1981

Modified from Long and Achterberg 2011.

**Table:** 

1	Mesosoma flattened, scutellum at same level as propodeum; scutellum almost smooth and without a transverse posterior carina; hind coxa more or less shortened, not surpassing apex of tergite I	2
–	Mesosoma normal (Figs 1A–F), scutellum protruding far above level of propodeum; scutellum rugose or punctate-rugose or finely punctate, often with an apical spine; hind coxa long, distinctly surpassing apex of tergite I	3
2	Propodeum with a very coarse median carina combined with various strong secondary rugae; body large (about 5.5 mm), completely brownish yellow. [Distribution. Papua New Guinea: East New Britain (Kerevat)]	*Wilkinsonellus daira* (Nixon, 1965)
–	Propodeum with a coarse median carina dividing propodeum into two smooth lateral parts, without rugae; body rather small (2.4 mm), black, metasoma yellowish brown, tergite I ivory colored laterally. [Distribution. North of Vietnam (Ha Tay)]	*Wilkinsonellus flavicrus* Long & van Achterberg, 2011
3	Ocelli small or medium-sized (Figs 3B, 4B, 5B), OOL more than diameter of posterior ocellus or subequal, inner margins of eyes at antennal sockets hardly or not emarginate	4
–	Ocelli large to very large (Fig. 2E), OOL less than diameter of posterior ocellus or equal; inner margins of eyes at antennal sockets deeply emarginate	16
4	Mesopleuron obliquely striate above precoxal sulcus; OOL 1.0–1.5 times diameter of posterior ocellus. [Distribution. Australia: Queensland (Atherton, Little Mulgrave Natl Pk, Mt Webb Natl Pk). Papua New Guinea: Western Highlands (Baiyer river Sanctuary), Morobe (Mt Keinde [Kaindi], Sattelberg)]	*Wilkinsonellus striatus* Austin & Dangerfield, 1992
–	Mesopleuron smooth or sparsely punctate above precoxal sulcus; OOL 1.6–2.0 times diameter of posterior ocellus	5
5	Body completely brownish yellow (Figs 1E–F, 4A, 5A)	6
–	Body partly dark brown or blackish, at least propodeum and mesopleuron (Figs 1D, 3A)	10
6	Face coarsely reticulate-rugose	7
–	Face finely and densely punctate	8
7	Body entire fulvous, except hind femur and hind tibia slightly darkened at extreme apex; OOL less than twice diameter of posterior ocellus. [Distribution. Philippines: Mindanao (Davao). Taiwan: Pingtung (Kenting Ntl. Pak., Shantimen [Sandimen?])]	*Wilkinsonellus iphitus* (Nixon, 1965)
–	Body yellowish orange except tergites III –IV (medially) and following tergites dark brown, and hind tarsi infuscate; OOL 1.27 times diameter of posterior ocellus. [Distribution. Reunion Island: Bras-Panon (La Caroline), Saint Pierre (Bassin Martin)]	*Wilkinsonellus narangahus* (Rousse & Gupta, 2013)
8	Hind wing with vannal lobe of typical microgastrine dimensions (Figs 3J, 5J). [Distribution. Fiji: Kavadu, Taveuni and Viti Levu]	*Wilkinsonellus nescalptura* Arias-Penna, Zhang & Whitfield, sp. n
–	Hind wing with vannal lobe reduced (Fig. 4J)	9
9	Outer dorsal surface of hind coxa with distinct longitudinal carina, inner dorsal surface coarsely reticulate; hypopygium smooth and hairless. [Distribution. Papua New Guinea: Jiwaka (Jimmi Valley), Madang, Morobe (Bulolo, Busu River in Lae, Lae-Zenag Road, Saruwaged Range), East New Britain (Kerevat). Australia: Queensland (MtTozer)]	*Wilkinsonellus tomi* Austin & Dangerfield, 1992
–	Outer dorsal surface of hind coxa with coarse and heterogeneous aerolate-rugose sculpture throughout without carina, ventral surface with dense and fine punctate those two areas separated by a flat, smooth and shiny stripe (Fig. 1B); hypopygium setose (Fig. 4H). [Distribution. Fiji: Gau, Kadavu, Taveuni, Vanua Levu, Viti Levu]	W. *fijiensis* Arias-Penna, Zhang & Whitfield, sp. n.
10	Scutellum with a small spine apically (Fig. 1F)	11
–	Scutellum without spine apically	15
11	Head yellow-orange or reddish-brown (Fig. 1D, 3A)	12
–	Head black or blackish brown	14
12	Frons with two distinct parallel carinae between antennal sockets. [Distribution: Central highlands of Vietnam (Kon Tum)]	*Wilkinsonellus nigrocentrus* Long & van Achterberg, 2011
–	Frons with rippled sculptures between antennal sockets (Fig. 3B)	13
13	Hind coxa reddish-brown; surface of hind coxa reticulate with fine granulate background sculpture. Head and mesosoma reddish-brown. [Distribution. India: Maharashtra (Solapur)]	*Wilkinsonellus granulatus* Ahmad, Pandey, Haider & Shuja-Uddin, 2005
–	Hind coxa completely black, but yellow-brown ventrally; outer dorsal surface of hind coxa with coarse aerolate-rugose sculpture, but finely sculptured in the remaining area (Fig. 1A); head yellow-orange and mesosoma brown-black. [Distribution. Fiji: Kadavu, Taveuni, Vanua Levu, Viti Levu]	*Wilkinsonellus corpustriacolor* Arias-Penna, Zhang & Whitfield, sp. n.
14	Vein 1CUa of fore wing 0.50 times as long as vein 1CUb; pterostigma distinctly shorter vein R1 (23: 60); frons smooth; propodeum largely rugose; vein cu-a of hind wing more or less sinuate. [Distribution. Vietnam: NE (Ha Giang), Central highlands (Lam Dong)]	*Wilkinsonellus nigratus* Long & van Achterberg, 2011
–	Vein 1CUa of fore wing 0.85 times as long as vein 1CUb; pterostigma as long as vein R1; frons rugose/punctate; propodeum sparsely rugose apically, smooth basally; vein cu-a of hind wing curved. [Distribution. Northeast of Vietnam (Thai Nguyen)]	*Wilkinsonellus masoni* Long & van Achterberg 2011
15	Head entirely, mesoscutum and scutellum black; frons, vertex and temple dull, coarsely rugose-punctate; scutellum medially dull and densely rugose; second metasomal tergite with an elongate parallel-sided area. Distribution. Belgian Congo (Nyasheke [Now Rwanda: Nyamasheke?]. Kenya (Embu)]	*Wilkinsonellus henicopus* (de Saeger, 1944)
–	Head brownish yellow, mesoscutum and scutellum reddish brown; frons, vertex and temple shiny and almost smooth; scutellum medially shiny and superficially rugose-punctate; second tergite without such area. [Distribution. Philippines: Luzon (Mt Makiling)]	*Wilkinsonellus thyone* (Nixon, 1965)
16	Lateral lobes of mesoscutum and mesopleuron ventrally yellow or brownish yellow; ocelli strongly protuberant, in frontal view completely above dorsal level of eyes	17
–	Lateral lobes of mesoscutum and mesosternum dark brown or blackish; ocelli less protuberant, in frontal view partly below dorsal level of eyes	21
17	Hind coxa yellow/orange without dark brown patch. [Distribution. Australia: Queensland (Herbert River in Ingham, Hope Vale Mission, Mt Spec, Mt Baird), Northern Territory (Mudginbarry H.S.)]	*Wilkinsonellus amplus* Austin & Dangerfield, 1992
–	Hind coxa yellow with apex light brown or with a dark brown ventral patch (Figs 1A–C)	18
18	Notauli absent. [Distribution. Vietnam: North Central Coast (Ha Tinh, Nghe An), Northeast (Thai Nguyen), Central highlands (Dak Lak), Southeast (Dông Nai)]	*Wilkinsonellus longicentrus* Long & van Achterberg, 2003
–	Notauli present but incomplete	19
19	Scutellar sulcus with seven carinate foveae. Axillary trough of metanotum with complete parallel carinae. Eyes and ocelli appearing reddish in preserved specimens (Figs 1C, 2E) [habitus Fig. 1C]. [Distribution. Panamá: Panamá (Las Cruces)]	*Wilkinsonellus panamaensis* Arias-Penna & Whitfield, 2013
–	Scutellar sulcus with five carinate foveae. Axillary trough of metanotum with some incomplete parallel carinae. Eyes and ocelli silver in preserved specimens	20
20	Fore and hind wings infuscate [habitus Fig. 1B]. [Distribution. Colombia: Magdalena (Tayrona Natl Pk), Chocó (Utría Natl Pk)]	*Wilkinsonellus kogui* Arias-Penna & Whitfield, 2013
–	Fore wing and hind wing not infuscate [habitus Fig. 1A]. [Distribution. Costa Rica: Alajuela (Area de Conservación Guanacaste)]	*Wilkinsonellus alexsmithi* Arias-Penna & Whitfield, 2013
21	Temple narrow, in lateral view its width near middle of eye 0.3–0.35 times transverse diameter of eye; OOL of female 0.2–0.3 times diameter of posterior ocellus; vertex without transverse rugosities. [Distribution. Vietnam: Northwest (Hoa Binh), Southeast (Dông Nai), Central highlands (Dak Lak), South central coast (Ninh Thuân)]	*Wilkinsonellus paramplus* Long & van Achterberg, 2003
–	Temple wider, in lateral view its width near middle of eye 0.4–0.5 times transverse diameter of eye; OOL of female 0.5 times diameter of posterior ocellus; vertex with distinct transverse rugosities. [Distribution. Vietnam: Northeast (Thai Nguyen), North Central Coast (Thua Thien-Hue)]	*Wilkinsonellus tobiasi* Long, 2007

### Descriptions of new species

#### Wilkinsonellus corpustriacolor

Arias-Penna, Zhang & Whitfield sp. n.

http://zoobank.org/45035C2E-F223-463F-BB64-2CA0DD2091E1

http://species-id.net/wiki/Wilkinsonellus_corpustriacolor

Figs 3A–K

##### Female.

Body length 2.2 mm, antennae length 2.73 mm.

##### Material examined.

**Type material.** Holotype, 1 female, FIJI: KADAVU ISLAND, Takuvi, 0.25 km SW Solodamu village, Moanakaka bird sanctuary, lat -19.078, long 178.121, 60 m, Malaise, coastal limestone forest, 07.iii–11.iv.2004, S. Lau, [FJKV41a].

Paratypes, 1 female, FIJI: VITI LEVU ISLAND, Nabukavesi village, Ocean Pacific Resort, lat -18.171, long 178.258, 40 m, Malaise, coastal lowland moist forest, 26.iv–05.v.2004, W. Naisilisili, [FJVL18a_01_25] in CAS. 1 male, 2 km E Navai village, old trail to mount Tomaniivi (Victoria), lat -17.621, long 178, 700 m, Malaise, gymnosperm dominated rainforest, 13–18.ii.2004, E. Namatalau, [FJVL11b_03_35] in California Academy of Sciences. 1 male & 1 female, FIJI: VITI LEVU ISLAND, 0.75 km E. Navai Village, old trail to mount Tomaniivi (Victoria), lat -17.621, long 177.989, 700 m, Malaise, gymnosperm dominated rainforest, 03.ii–16.iii.2005, E. Namatalau, [FJVL11d_05_26] in Bishop Museum.

##### Other material.

TAVEUNI ISLAND: 1 male, 3.2 km NW Lavena village, Mt. Koronibuabua, lat -19.851, long -179.891, 235 m, Malaise, lowland rainforest, 07.iii-11.iv.2004, B. Soroalau, [FJTA52d].

VANUA LEVU ISLAND: 1 male, Lomaloma village, lat -16.63, long -179.208, 587 m, Malaise, 26.i–07.ii.2006, N. Qarau, [FJVN97_03_01]; 1 male, same data except for: 630 m, 07–18.ii.2006, [FJVN95_01_02].

VITI LEVU ISLAND: 1 male, 2 km E Navai village, old trail to mount Tomaniivi (Victoria), lat -17.621, long 178, 700 m, Malaise, gymnosperm dominated rainforest, 13–18.ii.2004, E. Namatalau, [FJVL11b_03_35]; and 2 males, same data except for: Navai Village, 07–26.i.2004, [FJVL11e_01_02]. 1 male, 1.1 km SSW Volivoli village, Sigatoka sand dunes, lat -17.621, long 177.989, 55 m, Malaise, mixed littoral forest on sand, 06–17.iv.2004, S. Niusoria, [FJVL6b_02_19]; 1 female same data except for: 24.xi–15.xii.2003, T. Ratawa, [FJVL6b_02_16] and 1 female same data except 0.8 km SSW Volivoli village, 25 m, 24.xi–15.xii.2003, T. Ratawa, [FJVL6a_01_11].

##### Diagnosis.

Head yellow-orange, metasoma light yellow, mesosoma brown-black (Fig. 3A). Hind coxa brown-black, but yellow-brown ventrally and differing in coloration from fore and middle coxae; hind coxa with aerolate-rugose sculpture which varies in size and shape: outer dorsal edge coarse and heterogeneous, but fine and homogeneous in the remaining area (Fig. 2A). Proximal five antennal flagellomeres lighter than following flagellomeres. Ovipositor sheaths brown (Fig. 3K). Fore wing with vein r slightly curved (Fig. 3I). Hind wing with vannal lobe not reduced (Fig. 3J). Petiole of tergite I smooth, bottle-shaped, widest part with more or less straight edges (Fig. 3K).

##### Description.

Coloration (Figs 3A–K). Head yellow-orange, metasoma light yellow and mesosoma brown-black (Fig. 3A) except propleuron (Fig. 3G), dorsal epicnemial ridge and mesosternum yellow-brown. Legs: fore and middle coxa, and trochanter and trochantellus of all legs light yellow (Fig. 3A); hind coxa brown-black except ventrally yellow-brown (Fig. 2A), half basal of hind tibia and whole hind tarsus light brown. Proximal five antennal flagellomeres lighter than remaining brown flagellomeres; scape and pedicel light yellow, both with a thin brown strip laterally (Fig. 3D). Eyes and ocelli silver in preserved specimens, ocellar triangle area with a thin semicircular black ring in each ocellus (Fig. 3B). Edges of mandibular teeth brown. Membrane and microtrichiae of both fore and hind wings light brown (Figs 3I–J). Ovipositor sheaths brown (Fig. 3K).

Head (Figs 3B–D). Inner margin of scape curved, scape longer than wide (0.15:0.10 mm); pedicel as long as wide (0.06:0.07 mm), first three antennal flagellomeres equal in length (0.17:0.17:0.17 mm); last antennal flagellomerus with apex acute and 1.6× longer than penultimate (0.19:0.12 mm) flagellomere. Antennal scrobes deep, not encircled by carina and located far above middle level of eyes and close to inner compound eye margin; frons rippled sculptures throughout; in frontal view, medial area between antennal sockets without projection. Face with finely, dense and homogeneous punctures, interspaces wavy; face with a short median longitudinal carina running from antennal scrobes to half of the length of the face, but it continues as a ridge extending close to the clypeus; fronto-clypeal suture absent. Distance between anterior tentorial pit and closest inner compound eye margin 1.7× longer than diameter of tentorial pit (0.05:0.03 mm); anterior tentorial pits (Fig. 3C) far away from each other (0.14 mm). Mandible with two teeth, inferior tooth longer than superior. Maxillary palps longer than labial palps. OOL (Fig. 3B) 1.8× longer than diameter of lateral ocellus (0.11:0.06 mm), POL (Fig. 3B) subequal as diameter of lateral ocellus (0.07:0.06 mm). Vertex pointed, laterally sloped, but medially high (Fig. 3D), medially vertex with a semicircular, smooth and slightly concave area. Vertex and gena with fine, dense and homogeneous punctures, interspaces forming faint wavy patterns (Fig. 3B).

Mesosoma (Figs 3E–G). Mesosoma dorsoventrally convex (Fig. 3G). Pronotum shiny, smooth; curvature of pronotum with a deep groove of deeply carinate foveae throughout. Mesopleuron (Fig. 3G) smooth, convex, except precoxal groove with elongated foveae; margins lateral and ventro-lateral of mesopleuron forming an L-shaped area, ventro-lateral part with distinctive diagonal costate and lateral margin with a noticeable curved carina; dorsal epicnemial ridge convex. Mesosternum slightly flat with characteristic curved costate sculptures, medially with a groove of deep, carinate foveae. Metepisternum and metepimeron (Fig. 3G) separated by a chain of deep foveae, the deepest fovea at the dorsal end; metepisternum narrower than metepimeron, anteriorly metepisternum with a curvilinear carina running parallel to the groove of foveae that sorted it from the mesopleuron; metepisternum smooth but setose on dorsal edge; metepimeron just above submetapleural carina with finely areolate-rugose and hairs extended over most of the area. Mesoscutum (Fig. 3E) with dense areolate-rugose sculptures. Notauli (Fig. 3E) incomplete, not reaching the scutellar sulcus, barely visible by a slight difference in the level of the surface of the mesoscutum. Area of mesoscutum close to scutellar sulcus smooth and sloped. Scutellar sulcus (Fig. 3F) with at least six-seven deep, carinate foveae of heterogeneous size. Scutellum (Figs 3E–F) with sculptures of the same kind as mesoscutum and contours carinate; in lateral view scutellum with a spine apically (Fig. 2F). ATS (Fig. 3F) with several carinae; ATM (Fig. 3F) smooth with some short stubs only at posterior edge; L (Fig. 3F) smooth and shiny; BS (Fig. 3F) with tiny sculptures, dorsal edge upward; MPM (Fig. 3F) triangular, which apex strongly upward forming a carinate projection; BM (Fig. 3F) convex; PRM (Fig. 3F) thin, wavy and smooth. Propodeum (Fig. 3F) with a complete median longitudinal carina dividing it in two halves, plus one additional divergent carina at each half of propodeum; all three carinae crossed by several transverse, wavy carinae; edge of first third anterior of propodeum with less transverse carinae. Propodeal spiracle enclosed partially by carinae, but anteriorly without evident transverse carina; propodeal spiracle located at the intersection between pleural and a posterior transverse carina; inner spiracle far away from divergent carina (Fig. 3F).

Wings (Figs 3I–J). Fore wing with vein r slightly curve (0.15 mm) arising just beyond the half of the length of pterostigma (Fig. 3I); vein 2RS as same length as r (0.15:0.15 mm), but 2.1× longer than 2M and (RS+M)b veins (0.15:0.07:0.07 mm). Hind wing (Fig. 3J) with vannal lobe of normal size but with subapical outline flattened; edge with setae throughout, basal ones longer than apical.

Legs. Hind coxa extended beyond apex of tergite III (Fig. 3A) with aerolate-rugose sculptures, but differing in size and shape: outer dorsal edge coarse and heterogeneous, but fine and homogeneous in the remaining area (Fig. 2A); hind tibia with inner spur 1.3× longer than outer spur (0.22:0.17 mm); hind basitarsus 1.8× longer than inner spur (0.40:0.22 mm), telotarsus subequal in length to penultimate tarsomere (0.11:0.10 mm); outer surface of hind tibia with orderly spines throughout.

Metasoma (Figs 3H, K). Petiole of tergite I (Fig. 3K) smooth, bottle-shaped widest part with mildly straight edges, length 0.35 mm, distinctly constricted at anterior half (minimum width 0.04 mm), but subapically wider (maximum width 0.09 mm), petiole with a deep groove which reaches the half of the length of swollen area; hypopygium not protruding beyond at apex of metasoma (Fig. 3H); wall of hypopygium with long and numerous hairs; ovipositor length = 0.10 mm, apex rounded and bearing few long hairs, in lateral view ovipositor sheaths not protruding beyond the apex of metasoma.

Comments. In some females from Viti Levu and Kavadu, at least the first five antennal flagellomeres are lighter than the remaining ones. Some females have the ocellar triangle black, hiding the semicircular ring in each ocellus. Tergite III and beyond with some brown tinge. Body length range from 2.22 to 2.93 mm.

Males. Males from all localities exhibit tergites II and beyond brown in comparison with females. In some specimens, basal antennal flagellomeres look similar in coloration to apical flagellomeres, and ocellar triangle with extended black area, so black ring around each ocelli is not outlined. Body length range from 2.2 to 2.52 mm.

##### Etymology.

From *corpus* (Latin, noun) = body; *tres, tria* (Latin, number) = three and *color*, *colos* (Latin, noun)= color, tint, hue. The name refers to different coloration on the body: head, mesosoma and metasoma.

##### Distribution.

Fiji: Kadavu, Taveuni, Vanua Levu and Viti Levu. *Wilkinsonellus corpustriacolor* sp. n was collected in coastal limestone forest, coastal lowland moist forest, lowland rainforest, and gymnosperm dominated rainforest and elevation ranges from 25 m to 700 m.

##### Host.

unknown

#### Wilkinsonellus fijiensis

Arias-Penna, Zhang & Whitfield sp. n.

http://zoobank.org/2DA0845E-CFCD-40D8-B5E8-3B2D814DC31B

http://species-id.net/wiki/Wilkinsonellus_fijiensis

Figs 4A–K

##### Female.

Body length 2.63 mm, antennae length 3.33 mm

##### Material examined.

**Type material.** Holotype, 1 female, FIJI: VITI LEVU ISLAND, 2 km E Navai village, old trail to mount Tomaniivi (Victoria), lat -17.612, long 178, 700 m, Malaise, gymnosperm dominated rainforest, 13–18.ii.2004, [FJVL11b_03_35]. Paratypes, 1 female, same data as holotype in California Academy of Sciences. 1 male in California Academy of Sciences and 1 female & 1 male in Bishop Museum, same data as holotype except for: 0.75 km E. Navai village, old trail to mount Tomaniivi (Victoria), lat -17.62, long 177.989, 03.ii–16.iii.2005, [FJVL11d_05_26].

##### Other material.

GAU ISLAND: 1 female, 4 km SE Navukailagi village, mount Delaco, lat -17.98, long 179.275, 496 m, Malaise, 28.xii.2005–10.i.2006, U. Racule, [FJGA65_02_22]; 1 female, same data except for: 10.i–11.ii.2006, [FJGA65_02_23]; 1 female, same data except for: 3.3 km SE Navukailagi village, mount Delaco, 564 m, 20.x–02.xi.2005, [FJGA66_03_19].

KADAVU ISLAND: 1 male, Takuvi, 0.25 km SW Solodamu village, Moanakaka bird sanctuary, lat -19.078, long 178.121, 60 m, Malaise, coastal limestone forest, 23.x–19.xii.2003, S. Lau, [FJKV41a_02_06]; 3 males, same data except for: 128 m, 11.vi–06.vii.2003, [FJKV41b_01_02]; 1 male, same data except for: 128 m, 23.x–19.xii.2003, [FJKV41c_01_01].

TAUVENI ISLAND: 3 females, 5.6 km SE Tavuki village, Devo peak, lat -16.8432, long -179.965, 1187 m, Malaise, cloud forest, 03-10.i.2003, E. Ratu, [FJTA8a_01_12]; 1 female, Tavuki village, mount Devo, lat -16.837, long -179.973, 892 m, Malaise, montane wet forest, 31.vii-14.viii.2004, P. Vodo, [FJTA9b_04_02] in FNCI. 2 males & 4 females, Tavuki village, Devo peak, Malaise, M. Irwin, E. Schlinger & M. Tokotaa, 10-16.i.2003 [FJTA7-9]. 1 male, 3.2 km NW Lavena village, mount Koronibuabua, lat -16.855, long -179.889, 219 m, Malaise, lowland rainforest, 04–19.xi.2003, [FJTA52b_04_26].

VANUA LEVU ISLAND: 1 female, 6 km NW Kilaka, village Batiqere range, lat -16.8103, long 178.988, 61 m, Malaise, lowland wet forest, 03-10.vi.2004, P. Manueli, [FJVN58a_03_06]; 1 female, same data except for: lat -16.806, long 178.991, 98 m, 15-24.vi.2004, [FJVN58b_05_07]; 1 female, same data except for: alt -16.806, long 178.988, 154 m, [FJVN58e_04_06]; 3 males, same data except for: -16.806, long 178.988, 154 m, 28.vi-02.vii.2004, [FJVN58e_04_08]. 1 female, Lomaloma village, 630 m, Malaise, 07–18.ii.2006, N. Qarau, [FJVN95_01_02]; 1 female, same data except for: 219 m, 26.i–07.ii.2006, [FJVN96_02_01]; 1 male & 2 females, same data except for: 587 m, 26.i–07.ii.2006, [FJVN97_03_01]; 2 females, same data except for: 587 m, 07-18.ii.2006, [FJVN97_03_02].

VITI LEVU ISLAND: 1 male, 1 km E Abaca village, mount Evan’s range, Koroyanitu Eco park, Kokabula trail, lat -17.667, long 177.55, 800 m, Malaise, disturbed mid-elevation moist forest, 26.xi–03.xii.2002, L. Tuimereke, [FJVL02_01_09]; 3 males, same data except: 0.5 km E Abaca village, [FJVL03_01_09]; 2 males, same information except: 0.5 km E Abaca village, 12–19.xi.2002 [FJVL03_01_54]. 1 male, 4 km WSW Colo-i-Suva village, mount Nakobalevu, lat -18.055, long 178.423, 372 m, Malaise, lowland wet forest, 12–24.vi.2004, Timoci, [FJVL4b_03_33]. 1 female, 0.8 km SSW Volivoli village, Sigatoka sand dunes, lat -18.166, long 177.485, 4 m, Malaise, mixed littoral forest on sand, 24.xi–15.xii.2003, T. Ratawa, [FJVL6a_01_11]; 1 male & 4 females, same data except for: 1.1 km SSW Volivoli village, lat -18.171, long 177.484, 700 m, 09–20.xii.2003 [FJVL6c_04_11]. 1 female, 1.8 km E Navai village, old trail to mount Tomaniivi (Victoria), lat -17.621, long 177.998, 700 m, Malaise, gymnosperm dominated rainforest, 24.x-08.xi.2003, E. Namatalau, [FJVL11c_04_08]; 2 males, same data except for: Navai village, old trail to mount Tomaniivi (Victoria), lat -17.616, long 177.983, 07–26.i.2004, [FJVL11e_01_02]. 1 female, 3.3 km N Veisari settlement, logging road to Waivudawa, lat -18.069, long 178.3666, 300 m, Malaise, lowland wet forest, 08–31.iii.2003, M. Tokotaa [FJVL10d_04_02]. 2 males & 1 female, 3.2 km E Navai village, Veilaselase Track, lat -17.624, long 178.009, 700 m, Malaise, gymnosperm dominated rainforest, 06.vi–15.vii.2003, E. Namatalau, [FJVL11a_02_02]. 1 male, Nabukavesi Village, Ocean Pacific Resort, lat -18.171, long 178.258, 40 m, Malaise, coastal lowland moist forest, 26.iv–05.v.2004, W. Naisilisili, [FJVL18a_01_25].

##### Diagnosis.

Propodeal spiracles touching the pleural carina and enclosed by an incomplete carinate area (Fig. 4F). Hind coxa (Fig. 2B) with three distinctive regions: outer dorsal surface with big and heterogeneous aerolate-rugose throughout; ventral surface with dense, fine punctate; those two kind of sculptures separated by a flat, smooth and shiny stripe. Petiole (Fig. 4K) bottle-shaped, widest part with edge not strongly curved. Axillary trough of scutellum (Fig. 4F) with fine, dense and homogeneous punctures, interspaces wavy, forming undulating patterns. Fore wing (Fig. 4I) with vein r slightly curve. Hind wing (Fig. 4J) with vannal lobe reduced.

##### Description.

Coloration (Figs 4A–K). General body yellow (Fig. 4A). All antennal flagellomeres brown; scape (Fig. 4D) yellow with a brown strip in lateral outer surface; pedicel brown. Eyes and ocelli silver in preserved specimens, ocellar triangle (Fig. 4B) with semicircular black rings around each ocellus. Edge of mandibular teeth brown. Hind leg with both outer and inner spurs and all tarsomeres yellow-brown to brown. Tergites II and III (Fig. 4K) medially dark yellow-brown but lighter in periphery; tergite IV and so forth (Fig. 4K) brown, but subapically with a transversal thin yellow-brown band. Membrane and microtrichiae of both fore and hind wings light brown (Figs 4I–J).

Head (Figs 4B–D). Inner margin of scape curved, scape longer than wide (0.18:0.11 mm); pedicel as wide as long (0.05:0.06 mm), first three antennal flagellomeres subequal in length (0.20:0.21:0.21 mm). Antennal scrobes (Fig. 4B) deep, not surrounded by carina, located far above middle level of eyes (Fig. 4C); frons smooth but with some semicircles close to antennal sockets; in frontal view, medial area between antennal sockets without projection, antennal scrobes close to inner eye margin. Face (Fig. 4C) with fine, dense and homogeneous punctures, interspaces forming dorsally distinctive semicircular patterns which are less pronounced ventrally-close to the clypeus; face with a short noticeable median longitudinal carina running from antennal scrobes to half of the length of the face, but continuing as ridge extending close to clypeus; fronto-clypeal suture absent. Distance between each anterior tentorial pit and closest inner compound eye margin equal to diameter of a tentorial pit (0.05:0.04 mm); anterior tentorial pits far away from each other (0.15 mm). Mandible with two teeth, inferior tooth longer than superior. Maxillary palps longer than labial palps. OOL (Fig. 4B) 2× longer than diameter of lateral ocellus (0.15:0.07 mm), POL (Fig. 4B) shorter than diameter of lateral ocellus (0.04:0.07 mm). Vertex convex, laterally sloped and medially high, with fine, dense and homogeneous punctures, interspaces forming distinctive semicircular patterns close to the ocellar triangle and occiput, but less evident laterally; medially vertex with a smooth, shiny and slightly concave area. Gena with fine homogeneous punctures, interspaces forming wavy patterns close to occipital foramen.

Mesosoma (Figs 4E–G). Mesosoma dorsoventrally convex (Fig. 4G). Pronotum shiny, smooth; curvature of pronotum with elongated, semicircular and carinate foveae throughout the groove. Mesopleuron (Fig. 4G) convex, except precoxal groove which bears fine foveae; lateral and ventro-lateral margins forming a L-shaped area that possesses fine, homogeneous punctuations; lateral margin delimited by a carina; dorsal epicnemial ridge convex. Mesosternum slightly flat with dense, fine and homogeneous punctuations, medially with distinctive groove of deep, homogeneous foveae. Metepisternum and metepimeron (Fig. 4G) separated by a chain of deep foveae throughout, the largest at the dorsal end; metepisternum smooth and narrower than metepimeron; metepimeron just above submetapleural carina with very coarse areolate-rugose sculpture, remaining area without sculpturing. Mesoscutum (Fig. 4E) with fine, dense and homogeneous punctures, interspaces wavy forming transversal undulant patterns; mesoscutum slightly narrow than head. Notauli (Fig. 4E) incomplete, barely visible only in a small portion of the mesoscutum, faintly indicated by a change in sculpturing. Area of mesoscutum close to scutellar sulcus smooth and sloped. Scutellar sulcus (Figs 4E–F) with six deep, carinate foveae of heterogeneous size. Scutellum with the same kind of sculptures as mesoscutum and edges defined by a strong carina. ATS (Fig. 4F) with the same sort of sculpture as scutellum, without complete parallel carinae, and posterior edge with visible, but short stubs; ATM (Fig. 4F) with a few, incomplete parallel carinae, only present basally; L and BS (Fig. 4F) smooth and shiny; MPM (Fig. 4F) trapezoidal, and posteriorly delimited by a strong upward carina forming a projection; BM convex and PRM (Fig. 4F) thin and smooth. Propodeum (Fig. 4F) with a complete median longitudinal carina dividing it in two halves, plus one additional carina at each half of propodeum, all three carinae crossed by several transverse and wavy carinae. Propodeal spiracles touching the pleural carina and enclosed by an incomplete carinate area.

Wings (Figs 4I–J). Fore wing (Fig. 4I) with vein r slightly curved (0.15 mm) arising just beyond the half of the length of pterostigma; vein 2RS slightly longer than r (0.17:0.15 mm), but 1.8× longer than 2M and 1.5× longer than r(RS+M)b veins (0.17:0.09:0.11 mm). Hind wing (Fig. 4J) with vannal lobe reduced.

Legs. Hind coxa extending beyond apex of tergite III (Fig. 4A), hind coxa (Fig. 2B) with three distinctive regions: outer dorsal surface with coarse heterogeneous aerolate-rugose sculpturing throughout; ventral surface with dense, fine punctuates; those two kind of sculptures separated by a flat, smooth and shiny stripe; hind tibia with inner spur 1.6× longer than outer spur (0.36:0.23 mm); hind basitarsus 1.25× longer than inner spur (0.45:0.36 mm); outer surface of hind tibia with orderly spines throughout; hind tarsal claw with a short comb.

Metasoma (Figs 4H, K). Petiole of tergite I (Fig. 4K) smooth, bottle-shaped, widest part with more or less straight edges, length 0.35 mm, distinctly constricted at anterior half (minimum width 0.04 mm), but subapically wide (maximum width 0.10 mm), petiole with a deep groove extending ¾ the length of tergite I, just reaching the top of petiole widest part; hypopygium (Fig. 4H) not protruding at apex of metasoma; wall of hypopygium with long numerous hairs; ovipositor sheath length 0.06 mm, apex rounded and bearing tiny, few visible hairs, in lateral view ovipositor sheaths slightly protruding apex of metasoma.

Comments. In lateral view the mesosoma in some females (e.g. Viti Levu) exhibit two different shades of yellow, dorsally darker than ventrally. In specimens from Gau the coloration on tergite II and so forth is dark brown, in comparison with specimens from other sites that is light brown. Some females exhibit a black ocellar triangle area without/faint delimitation of semicircular black ring in each ocelli (e.g. Vanua Levu). Body length in females ranges from 2.02 mm to 2.83 mm.

Males. Similar to females. Some males exhibit a black ocellar triangle area without semicircular rings patterns in each ocellus (e.g. Kadavu). In specimens from Tauveni, Vanua Levu last laterotergites and sternites are brownish. Body length ranges from 2.22 mm to 2.93 mm.

##### Etymology.

The name is based on the country Fiji, where the holotype was collected; the species is recorded in several localities in the archipelago.

##### Distribution.

Gau, Kadavu, Taveuni, Vanua Levu and Viti Levu islands. *Wilkinsonellus fijiensis* sp. n was collected in different ecosystem as coastal limestone forest, coastal lowland moist forest, mixed littoral forest on sand, lowland rainforest, lowland wet forest, cloud forest, montane wet forest, gymnosperm dominated rainforest, disturbed mid-elevation moist forest. Elevation in localities ranges from 4 m to 1200 m.

##### Host.

unknown

#### Wilkinsonellus nescalptura

Arias-Penna, Zhang & Whitfield sp. n.

http://zoobank.org/6CC317A2-CB95-4F47-B901-96E1AC74AB16

http://species-id.net/wiki/Wilkinsonellus_nescalptura

Figs 5A–K

##### Female.

Body length 2.63 mm, antennae length 2.93 mm

##### Material.

**Type material.** Holotype, 1 female, FIJI: KADAVU ISLAND, Takuvi, 0.25 km southwest Solodamu village, Moanakaka bird sanctuary, lat- 19.077, long 178.121, 60 m, Malaise, coastal limestone forest, 07.iii–11.iv.2004, S. Lau, [FJKV41a]. Paratypes, 1 female & 1 male in California Academy of Sciences and 1 female & 1male in Bishop Museum, same data as holotype except for: 23.x–19.xii.2003, [FJKV41a_02_06].

##### Other material.

KADAVU ISLAND: 5 males & 1 female, same data as holotype except for: 23.x–19.xii.2003, [FJKV41a_02_06]; 2 males & 2 females, same data as holotype except for: 09–15.ii.2004, [FJKV41a_04_08].

TAUVENI ISLAND: 1 male & 1 female, 3.2 km NW Lavena village, mount Koronibuabua, lat -16.855, long -179.888, 219 m, Malaise, lowland rainforest 04–19.xi.2003, [FJTA52b_04_26]; 1 female, same data except for: lat -16.854, long -179.891, 235 m, 24.x–19.xi.2004, [FJTA52d]; 1 female, same data except for: lat -16.854, long -179.891, 235 m, 24.x-04.xi.2003, [FJTA52d_01_03]; 1 male, same data except for: lat -16.855, long -179.888, 229 m, 19.xi-19.xii.2003, [FJTA52f_05_28].

VITI LEVU ISLAND: 1 male, 1 km E Abaca village, mount Evan’s range, Koroyanitu Eco Park, Kokabula trail, lat -17.66, long 177.55, 800 m, Malaise, disturbed mid-elevation moist forest, 02–10.vi.2002, L. Tuimereke, [FJVL02_01_26]. 1 female, 2 Km SE Nabukavesi village, ocean Pacific resort, lat -18.170, long 178.258, 40 m, Malaise, coastal lowland moist forest, 26.iv–05.v.2004, W. Naisilisili, [FJVL18a_01_25].

##### Diagnosis.

First five antennal flagellomeres lighter in color than following flagellomeres. ATS and ATM smooth (Fig. 5F). Mesosternum with characteristic curved costate sculptures. Hind coxa (Fig. 2C) aerolate-rugose, sculptures on the outer dorsal edge big and heterogeneous, but fine and homogeneous in the remaining area. Fore wing (Fig. 5I) with vein r straight; hind wing (Fig. 5J) with vannal lobe normal, of typical microgastrine dimensions. Petiole of tergite I (Fig. 5K) bottle-shaped, widest part with rounded edges. Ovipositor sheaths brown (Fig. 5H).

##### Description.

Coloration (Figs 5A–K). General body (Fig. 5A) yellow-brown, except first five antennal flagellomeres yellow-brown, but remaining brown; lateral surface of both scape (Fig. 5D) and pedicel with a thin brown strip. Eyes and ocelli silver in preserved specimens; ocellar triangle with a slim semicircular black ring in each ocellus (Fig. 5B). Edges of mandibular teeth brown. Coxae of both front and middle legs, trochanter and trochantellus of all legs yellow (Fig. 5A). Petiole on tergite I (Fig. 5K) completely dark yellow, but the tergite I light yellow; median area in tergite II dark yellow with contours dark brown, coloration decreases in intensity as it gets far away from median area; tergites III and beyond brown; laterotergites and sternites yellow (Fig. 5H). Membrane and microtrichiae of both fore and hind wings light brown (Figs 5I–J). Ovipositor sheaths brown (Fig. 5H).

Head (Figs 5A–D). Inner margin of scape curved, scape longer than wide (0.15:0.10 mm); pedicel longer than wide (0.08:0.05 mm); first three antennal flagellomeres subequal in length (0.21:0.20:0.19 mm); last antennal flagellomerus longer than penultimate (0.20:0.14 mm) and with acute apex. Antennal scrobes (Figs 5B–C) shallow, not surrounded by carina located far above middle level of eyes and close to inner compound eye margin; frons with ripples sculptures throughout; in frontal view, medial area between antennal sockets without projection (Fig. 5C). Face with fine, dense and homogeneous punctures, interspaces forming dorsally distinctive semicircular patterns; face with a short median longitudinal carina running from antennal scrobes to half of the length of the face, but continuing as a ridge extending close to the clypeus; fronto-clypeal suture absent. Distance between anterior tentorial pit and closest inner compound eye margin 1.7× longer than diameter of tentorial pit (0.05:0.03 mm); anterior tentorial pits far away from each other (0.18 mm). Mandible with two teeth, inferior tooth longer than superior. Maxillary palps longer than labial palps. OOL (Fig. 5B) 1.7× longer than the diameter of lateral ocellus (0.12:0.07 mm), POL (Fig. 5B) as equal as diameter of lateral ocellus (0.07:0.07 mm). Vertex convex, laterally sloped and medially high, with fine, dense and homogeneous punctures, interspaces forming distinctive semicircular patterns; medially vertex with a smooth and slightly concave area. Gena (Fig. 5D) with fine homogeneous punctures, interspaces forming wavy patterns.

Mesosoma (Figs 5E–G). Mesosoma dorsoventrally convex (Fig. 5G). Pronotum shiny, smooth; curvature of pronotum (Fig. 5G) with a deep groove of deep foveae throughout. Mesopleuron (Fig. 5G) smooth, convex, except precoxal groove which bears a group of foveae; margins lateral and ventro-lateral of mesopleuron forming an L-shaped area; ventro-lateral part with distinctive diagonal costate sculpturing and lateral margin with an evident curved carina; dorsal epicnemial ridge convex. Mesosternum slightly flat with characteristic curved costate sculptures; medially with a groove of deep, homogeneous foveae. Metepisternum and metepimeron (Fig. 5G) separated by a chain of deep foveae, the deepest fovea at the dorsal end; metepisternum narrower than metepimeron; metepisternum with an additional curved carina running parallel to the groove of foveae that separates it from the mesopleuron; dorsal edge of metepisternum with a convex and setose area; metepimeron just above submetapleural carina with coarse areolate-rugose sculpturing, remaining area with scattered, finely sculpture, hairs extending over most of the area (Fig. 5G). Mesoscutum (Fig. 5E) with fine, dense and homogeneous punctures, interspaces wavy, mesoscutum slightly narrow than head. Notauli (Fig. 5E) incomplete, not reaching the scutelar sulcus, visible in most of the anterior part of the mesoscutum and indicated by a faint change in sculpturing. Area of mesoscutum close to scutellar sulcus smooth and sloped. Scutellar sulcus (Figs 5E–F) with at least six visible deep, carinate foveae of heterogeneous size. Scutellum with edges carinate, sharing the same kind of sculptures as mesoscutum. ATS and ATM (Fig. 5F) smooth, both with some short stubs at posterior edge; L and BS (Fig. 5F) smooth and shiny; MPM (Fig. 5F) trapezoidal, posteriorly with a strong upward carina forming a projection; BM convex and PRM (Fig. 5F) thin, wavy and smooth. Propodeum (Fig. 5F) with a complete median longitudinal carina, plus one additional divergent carina at each half of propodeum, all three carinae crossed by several transverse, wavy carinae; first third anterior of propodeum without transverse carinae. Propodeal spiracles in the junction of transverse carina and pleural carina; innerly spiracles far away from divergent carina; anteriorly lacking of any evident carinae. Propodeal spiracle partially enclosed by carinae.

Wings (Figs 5I–J). Fore wing (Fig. 5I) with vein r straight (0.16 mm) arising just beyond the half of the length of pterostigma; vein 2RS longer than r (0.20:0.16 mm), but 2× longer than 2M and r(RS+M)b veins (0.20:0.10:0.10 mm). Hind wing (Fig. 5J) with vannal lobe not reduced, but with subapical outline flattened; edge with setae throughout, basal ones longer than apical.

Legs. Hind coxa extended beyond apex of tergite III (Fig. 5A) with aerolate-rugose sculpturing, sculpture on the outer dorsal edge coarse and heterogeneous, but fine and homogeneous in the remaining area (Fig. 2C); hind tibia with inner spur 1.3× longer than outer spur half (0.26:0.20 mm); hind basitarsus 1.5× longer than inner spur (0.40:0.26 mm), telotarsus as same length as penultimate tarsomere (0.10:0.10 mm); outer surface of hind tibia with orderly spines throughout.

Metasoma (Figs 5H, 5K). Petiole of tergite I (Fig. 5K) smooth, bottle-shaped, widest part with rounded edges, length 0.40 mm, distinctly constricted over anterior half (minimum width 0.05 mm), but subapically wider (maximum width 0.12 mm), petiole with a deep groove extending across great part of the petiole swollen area; hypopygium (Fig. 5H) not protruding beyond apex of metasoma; wall of hypopygium with long and numerous hairs; ovipositor sheath length 0.15 mm, apex acute, tapering at the ending, bearing long and visible hairs, in lateral view ovipositor sheaths not protruding the apex of metasoma.

Comments. In some females from Kavadu, the coloration on tergites III and beyond is completely yellow; other specimens exhibit on those tergites a striping pattern that can alternate light yellow followed by dark brown and vice versa. In contrast, specimens from Taveuni, the brown coloration includes tergite II and following. Body length ranges from 2.12 mm to 2.83 mm.

Males. Similar to females. Some dry specimens (e.g. from Kavadu) with striping pattern on tergites III and beyond, those specimens exposing the arthrodial membrane which is light yellow in contrast with dark brown coloration on the tergites. In other males, the first third anterior of propodeum lacks the transverse carinae. Body length ranges from 2.32 to 2.52 mm.

##### Etymology.

From *ne* (Latin)= particle of negation and *scalptura* (Latin, noun, femine) = engraving. The name refers at the absence of sculptures in both the axillary trough of the scutellum and axillary trough of the metanotum.

##### Distribution.

Kavadu, Taveuni and Viti Levu. *Wilkinsonellus nescalptura* sp. n was collected in coastal lowland moist forest, coastal limestone forest, lowland rainforest and disturbed mid-elevation moist forest, ecosystems range from 40 m to 800 m.

##### Host.

unknown

## Supplementary Material

XML Treatment for Wilkinsonellus

XML Treatment for Wilkinsonellus corpustriacolor

XML Treatment for Wilkinsonellus fijiensis

XML Treatment for Wilkinsonellus nescalptura
